# Genetic effects on educational attainment in Hungary

**DOI:** 10.1002/brb3.2430

**Published:** 2021-11-29

**Authors:** Péter P. Ujma, Nóra Eszlári, András Millinghoffer, Bence Bruncsics, Dóra Török, Péter Petschner, Péter Antal, Bill Deakin, Gerome Breen, György Bagdy, Gabriella Juhász

**Affiliations:** ^1^ Institute of Behavioural Sciences Semmelweis University Budapest Hungary; ^2^ National Institute of Clinical Neuroscience Budapest Hungary; ^3^ Department of Pharmacodynamics Faculty of Pharmacy Semmelweis University Budapest Hungary; ^4^ NAP‐2‐SE New Antidepressant Target Research Group Hungarian Brain Research Program Semmelweis University Budapest Hungary; ^5^ Department of Measurement and Information Systems Budapest University of Technology and Economics Budapest Hungary; ^6^ MTA‐SE Neuropsychopharmacology and Neurochemistry Research Group Hungarian Academy of Sciences Semmelweis University Budapest Hungary; ^7^ Division of Neuroscience and Experimental Psychology Faculty of Biology, Medicine and Health University of Manchester Manchester UK; ^8^ Manchester Academic Health Sciences Centre Manchester UK; ^9^ Greater Manchester Mental Health NHS Foundation Trust Manchester UK; ^10^ Social, Genetic and Developmental Psychiatry Centre King's College London London UK; ^11^ SE‐NAP 2 Genetic Brain Imaging Migraine Research Group Hungarian Brain Research Program Semmelweis University Budapest Hungary

**Keywords:** cognitive abilities, gene‐environment interaction, heritability, polygenic score, socioeconomic status

## Abstract

**Introduction:**

Educational attainment is a substantially heritable trait, and it has recently been linked to specific genetic variants by genome‐wide association studies (GWASs). However, the effects of such genetic variants are expected to vary across environments, including countries and historical eras.

**Methods:**

We used polygenic scores (PGSs) to assess molecular genetic effects on educational attainment in Hungary, a country in the Central Eastern European region where behavioral genetic studies are in general scarce and molecular genetic studies of educational attainment have not been previously published.

**Results:**

We found that the PGS is significantly associated with the attainment of a college degree as well as the number of years in education in a sample of Hungarian study participants (*N* = 829). PGS effect sizes were not significantly different when compared to an English (*N* = 976) comparison sample with identical measurement protocols. In line with previous Estonian findings, we found higher PGS effect sizes in Hungarian, but not in English participants who attended higher education after the fall of Communism, although we lacked statistical power for this effect to reach significance.

**Discussion:**

Our results provide evidence that polygenic scores for educational attainment have predictive value in culturally diverse European populations.

## Graphical abstract

We found that polygenic scores (PGS) associated with educational attainment (EA) in an international genome‐wide association study are also associated with EA in Hungary, despite historic, societal and economic differences between this country and those in which the PGS was discovered. Our results demonstrate the general validity of the PGS.

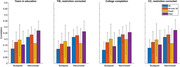

## 1 INTRODUCTION

Educational attainment is a key psychological and sociological variable, which comprises an important part of socioeconomic status and which is positively correlated with income and health, but negatively with crime and welfare dependency (Behrman et al., 1997). Educational attainment is moderately heritable, with a substantial shared environmental component (Branigan et al., 2013) and it shares substantial, but not all genetic variance with cognitive abilities (Krapohl et al., 2014).

Early reports on the heritability of educational attainment were derived from family pedigree studies, most notably twin studies (Cesarini & Visscher, 2017). Recently, however, the heritability of educational attainment was confirmed with molecular genetic methods. Single nucleotide polymorphism (SNP) heritability studies (Davies et al., 2016; Hill et al., 2016) confirmed that genetic similarity between non‐related individuals is positively associated with the phenotypic similarity of their educational attainment, with common genotyped SNPs accounting for up to 20% of the total variance. Over the past 10 years, a series of genome‐wide association (GWA) studies using a constantly expanding international study sample have been performed within the framework of the Social Science Genetic Association Consortium (SSGAC), linking specific genetic variants to educational attainment (Lee et al., 2018; Okbay et al., 2016; Rietveld et al., 2013). Polygenic scores (PGSs) based on GWAS results (referred to as EA1‐3 PGSs depending on which of the SSGAC GWAS results were used to construct them) confirmed the predictive value of these genetic variants (also termed PGS heritability), which typically account for up to 10% of the phenotypic variance in educational attainment itself (Allegrini et al., 2019; Domingue et al., 2015), cognitive abilities (Allegrini et al., 2019; de Zeeuw et al., 2014; Selzam et al., 2016), social mobility (Ayorech et al., 2017), and overall socioeconomic success (Belsky et al., 2018, 2016) in independent samples. The predictive performance of education attainment PGSs has been demonstrated among others in samples of Icelanders (Kong et al., 2018), Estonians (Rimfeld et al., 2018), and African Americans (Domingue et al., 2015; Lee et al., 2018; Rabinowitz et al., 2019).

However, neither the pedigree‐based or SNP heritability of educational attainment nor the correlation of polygenic scores with socioeconomic phenotypes is a biological constant. There is evidence that between‐country differences (Lee et al., 2018; Silventoinen et al., 2020) and within‐country changes in education policy (Heath et al., 1985), as well as the attendance of different types of schools (Trejo et al., 2018) may affect the heritability of educational attainment (gene‐environment interaction). In other words, the relative importance of genetic and environmental effects on individual differences in educational attainment is affected by the characteristics of the environment. It has been argued (Conley et al., 2015; Hauser, 2002; Nielsen, 2008) that a high heritability of educational attainment is a sign of a meritocratic educational system, because attainment is determined by innate abilities and preferences instead of shared environmental effects such as social class or parental income. The social changes due to the Fall of Communism (FoC) in the former Eastern Bloc may have had a particular effect on educational meritocracy. In line with this hypothesis, a recent Estonian study (Rimfeld et al., 2018) found that the SNP and PGS heritability of educational attainment was higher in Estonians who attended school after FoC, suggesting that the educational system in Estonia has become more meritocratic. In line with this observation, pedigree‐based studies conducted in countries with higher social mobility generally also show higher heritability (Engzell & Tropf, 2019).

Because of the moderating and mediating effects of environmental variables, the strength of genetic effects on educational attainment may be different across countries. In the present study, we investigated molecular genetic effects on educational attainment in Hungary, a country where no similar study has previously been published. The main purpose of the current study was to establish the presence and magnitude of the predictive performance of the latest educational attainment PGS in a Hungarian subsample. While the Hungarian population is not substantially genetically different from that of other European countries (Heath et al., 2008), the country is characterized by lower GDP, income, and according to some indicators, lower social mobility (Eurofound, 2017) compared to Western European countries where PGSs have been extensively used in research. Notably, the country transitioned from a planned economy to a market economy only about 15 years before our data was collected. These characteristics of Hungarian society and economy render it an interesting question to what extent the molecular genetic indicators discovered in other countries predict educational attainment, a key element of social and economic success, in Hungary.

As auxiliary analyses, we also estimated cohort differences in PGS heritability as well as overall SNP heritability. These are of interest because no data on these metrics is available from Hungary and we are unaware of any ongoing research to calculate these estimates from larger samples. We caution, however, that our study, while well powered for its main purpose, has limited statistical power to provide precise estimates of these latter effects.

Throughout the paper, we use the term “genetic effects” because the route of causation in this case can only go in one direction, from the genotype to the phenotype. However, we note that nominally genetic effects can be indirect and environmentally moderated (Young, 2019) in practice indexing environmental effects (see also Section 4).

## 2 MATERIAL AND METHODS

We used genetic data and self‐reported level of education collected in the NewMood study (New Molecules in Mood Disorders, Sixth Framework Program of the European Union, LSHM‐CT‐2004‐503474) to validate the EA3 polygenic score (Lee et al., 2018) in Hungarian participants (Budapest sample, *N* = 829). We used data from English participants from NewMood (Manchester sample, *N* = 976) to provide a comparison group with an identical phenotypic and genotypic data collection regimen. Participants of 18–60 years of age were recruited through advertisements, general practices, and a website. Full details of the recruitment strategy and criteria have been published previously (Juhasz et al., 2009, 2011; Lazary et al., 2008). NewMood was originally conceptualized as a study of psychiatric phenotypes and oversampled participants with psychiatric illness. Details about the incidence of psychiatric diagnoses and the results of symptom assessment scales (reported separately for the Budapest and Manchester samples) were reported previously (Eszlari et al., 2019), especially in Supplementary Table 1 of the referenced article. We addressed this limitation of the sample by adjusting models for self‐reported psychiatric and pain‐related disorders as covariates (see Section 3).

For this study, the experimental cohort was limited to unrelated individuals of self‐reported European white ancestry as this was the largest ethnic group.

The study was approved by the local Ethics Committees (Scientific and Research Ethics Committee of the Medical Research Council, Budapest, Hungary; and North Manchester Local Research Ethics Committee, Manchester, UK) and was carried out in accordance with the Declaration of Helsinki and all relevant rules and regulations as part of the NewMood study. All participants provided written informed consent.

### 2.1 Educational attainment

Participants filled out close‐ended questions about whether they attained certain educational levels. These levels were “No qualification,” “O‐levels,” “A‐levels,” “Degree,” “Professional qualification,” and “Other (please specify).” In the Hungarian version of the questionnaire, British educational levels were translated as their Hungarian counterparts (O‐levels as “szakmunkásképző,” vocational education; A‐levels as “érettségi,” high school diploma; professional qualification as “szakvizsga,” a vocational or specialist qualification). If a participant gave a response about an “Other” qualification, the participant was prompted to provide further detail about his/her qualification and an educational level was assigned based on this information.

We used these self‐reported educational attainment levels to create two educational attainment phenotypes. First, we coded whether each participant attained a tertiary degree (college completion). The choice of a simple binary phenotype was justified by the fact that most of the educational attainment variance was between tertiary degrees or the absence of them (Table 1). Second, we converted the self‐reported educational attainment levels to years of completed education as an interval variable (years in education). Years in education was imputed as the number of years in educational typically necessary to obtain the individual's qualification in the Hungarian system: 8 years for “no qualification,” 11 years for a professional vocational education (“szakvizsga”), 12 years for both a vocational and a standard high school (“szakmunkásképző,” “érettségi”), and 16 years for a university degree. Four extra years were added for a university degree as an average estimate because 3‐year and 5‐year postgraduate programs were pooled as an answer option (these programs have been replaced by BA/BSc and MA/MSc programs after data collection). If participants provided comments about the specifics of their qualification (e.g., 3‐year or 5‐year degree or a specific additional qualification), then the appropriate number of years in education was imputed. We used this imputation system in both the Budapest and Manchester samples to ensure compatibility. As a robustness check, we coded educational attainment as International Standard Classification of Education (ISCED) categories in an alternative model. Because participants attended school before the publication of the latest ISCED 2011 system, we used the more applicable ISCED 1997 system and inputted years in education as reported in the largest published GWAS (Lee et al., 2018). In this system, we inputted ISCED 1 (primary education, 7 years) for “no qualification,” ISCED 2 (lower secondary education, 10 years) for a professional vocational education, ISCED 3 (upper secondary education) for both vocational and standard high schools, but with 10 and 13 years of education, respectively, and ISCED 5 (tertiary education, 20 years) for university degrees. The original and the ISCED‐based years in education phenotype was highly correlated (*r* = 0.993 in the Budapest sample and *r* = 0.946 in the Manchester sample).

**TABLE 1:** The distribution of age and educational level across age groups

	**Budapest**				**Manchester**			
	YPostC	PostC	PreC	All participants	YPostC	PostC	PreC	All participants
**Education**								
Years in education (Mean)	12.18	13.68	13.98	13.25	14.08	14.11	13.38	13.71
Years in education (SD)	0.92	2.06	2.18	1.96	2.11	2.46	2.55	2.47
Mean age (SD)	20.98 (1.21)	27.95 (2.51)	42.65 (7.21)	30,77 (10.39)	20.5 (1.64)	27.96 (2.65)	42.17 (6.42)	33.94 (10.32)
No qualification	1 (0.36%)	1 (0.45%)	4 (1.43%)	6 (0.72%)	2 (1%)	5 (2.25%)	24 (4.35%)	31 (3.18%)
Professional qualification	1 (0.36%)	1 (0.45%)	1 (0.36%)	4 (0.48%)	5 (2.5%)	14 (6.31%)	70 (12.68%)	89 (9.12%)
O‐levels or equivalent	0 (0%)	10 (4.52%)	20 (7.17%)	36 (4.34%)	16 (8%)	50 (22.52%)	144 (26.09%)	211 (21.62%)
A‐levels or equivalent	259 (94.18%)	114 (51.58%)	109 (39.07%)	512 (61.76%)	70 (35%)	32 (14.41%)	89 (16.12%)	192 (19.67%)
University degree	14 (5.09%)	95 (42.99%)	145 (51.97%)	271 (32.69%)	107 (53.5%)	121 (54.5%)	225 (40.76%)	453 (46.41%)
All participants	275 (100%)	221 (100%)	279 (100%)	829 (100%)	200 (100%)	222 (100%)	552 (100%)	976 (100%)
**Age**								
18	5 (1.82%)	0	0	5 (0.65%)	23 (11.5%)	0	0	23 (2.36%)
19	25 (9.09%)	0	0	25 (3.23%)	45 (22.5%)	0	0	45 (4.62%)
20	65 (23.64%)	0	0	65 (8.39%	36 (18%)	0	0	36 (3.7%)
21	86 (31.27%)	0	0	86 (11.1%)	33 (16.5%)	0	0	33 (3.39%)
22	61 (22.18%)	0	0	61 (7.87%)	30 (15%)	0	0	30 (3.08%)
23	33 (12%)	0	0	33 (4.26%)	33 (16.5%)	0	0	33 (3.39%)
24	0	25 (11.31%)	0	25 (3.23%)	0	32 (12.4%)	0	32 (3.29%)
25	0	19 (8.6%)	0	19 (2.45%)	0	25 (9.69%)	0	25 (2.57%)
26	0	31 (14.03%)	0	31 (4%)	0	35 (13.57%)	0	35 (3.59%)
27	0	19 (8.6%)	0	19 (2.45%)	0	27 (10.47%)	0	27 (2.77%)
28	0	36 (16.29%)	0	36 (4.65%)	0	29 (11.24%)	0	29 (2.98%)
29	0	22 (9.95%)	0	22 (2.84%)	0	22 (8.53%)	0	22 (2.26%)
30	0	22 (9.95%)	0	22 (2.84%)	0	33 (12.79%)	0	33 (3.39%)
31	0	26 (11.76%)	0	26 (3.35%)	0	19 (7.36%)	0	19 (1.95%)
32	0	21 (9.5%)	0	21 (3.35%)	0	36 (13.95%)	0	36 (3.7%)
33‐35	0	0	52 (18.64%)	52 (2.71%)	0	0	79 (15.31%)	79 (8.11%)
35‐40	0	0	78 (27.96%)	78 (6.71%)	0	0	151 (29.26%)	151 (15.5%)
40‐45	0	0	53 (19%)	53 (10.06%)	0	0	145 (28.1%)	145 (14.89%)
45‐50	0	0	45 (16.13%)	45 (6.84%)	0	0	95 (18.41%)	95 (9.75%)
50‐55	0	0	36 (12.9%)	36 (5.81%)	0	0	18 (3.49%)	18 (1.85%)
55‐60	0	0	15 (5.38%)	15 (4.65%)	0	0	28 (5.43%)	28 (2.87%)
All participants	275 (100%)	221 (100%)	279 (100%)	775 (100%)	200	258	516	974

### 2.2 Age groups

We aimed to investigate whether the strength of genetic effects on educational attainment varied as a function of graduation cohort. First, cohorts were separated based on whether participants graduated from high school before or after FoC, a possible moderator of the relative strength of genetic effects at least in the Budapest sample (Rimfeld et al., 2018). A third category for very young participants (age <24 years) was split off from the PostC cohort because these participants were likely not to have completed their tertiary education regardless of their genetic endowment (see low educational attainment variability in Table 1) which would exert a downward bias on PGS heritability.

For ethical reasons, we did not store data about the birth year of our participants or exactly when they were interviewed. However, given that data collection was performed in 2004 and 2005, we can estimate the birth year of each participant within 1‐year margin based on self‐reported age at data collection. As described above, we divided our participants in three age groups based on their age at FoC and whether they were old enough to have realized their potential for educational attainment by the time data was collected: (1) YPostC (young participants attending high school after FoC): age <24 at data collection (earliest possible birth year in 1981, possibly not old enough to have attained a university education); (2) PostC (participants attending high school mostly after FoC): age 24–31 years at data collection (birth year 1973–1981, at most 16 years old at FoC in Hungary and old enough to have attained a university education); (3) PreC (participants attending high school mostly before FoC): age at least 32 years at data collection (latest possible birth year in 1973, at least 16 years old at FoC in Hungary). Alternative cohort cut‐off points were also explored (see Section 3).

We provide detailed statistics about the sample sizes, ages, and educational attainments of these groups in Table 1. We hypothesized that the predictive performance of the EA3 polygenic score will be different in these age groups in Hungary, but not in England, due to a gene‐environment interaction induced by the historical political changes in Hungary and their effects on the educational system (Rimfeld et al., 2018).

Note that the “All participants” columns under “Education” also contain participants with no age data, who were consequently not assigned to either age group (*N*_Budapest_ = 54, *N*_Manchester_ = 2). For the same reason, counts in these columns are not equal to the sum of the age group columns and the total count of “All participants” is different for the “Education” and “Age” panels.

### 2.3 Genotyping

Genomic DNA was extracted from buccal swabs collected by a cytology brush (Cytobrush plus C0012; Durbin PLC). Genomic positions were defined according to the build GRCh37/hg19. Individuals were genotyped using Illumina's CoreExome PsychArray yielding a total of 573,141 variants. Biallelic strand aligned autosomal SNPs were used for imputation. For haplotype information, SHAPEIT, and for the imputation, IMPUTE2 softwares were used. Multiallelic and not single‐nucleotide variants were excluded. Variants with an imputation score “info” less than 0.5 or “certainty” less than 0.7 were excluded. After that, variants and participants were filtered separately for each of the combined Budapest‐Manchester sample, the Budapest subsample, and the Manchester subsample. SNPs with minor allele frequency (MAF) less than 0.01 were excluded. SNPs and participants with missingness larger than 0.01 were excluded in an iterative process (0.1, 0.05, and 0.01). SNPs with *p*‐value ≤10^−5^ for the Hardy‐Weinberg equilibrium test were excluded. Individuals with a problematic inferred gender or outlying individuals based on heterozygosity were excluded. No pair of individuals with π^2^ ≥0.1875 was kept in the final dataset. For these latest steps of participant filtering and also for principal components analysis (PCA) of the genome, LD pruning was applied on the SNPs, with an *R* ^2^ threshold of 0.2 and a window of 1500 SNPs by 150 SNPs. In the PCA, the top 10 eigenvectors were extracted using the software tool EIGENSOFT (https://www.hsph.harvard.edu/alkes‐price/software/) and the smartpca procedure, which implements the algorithm described elsewhere (Patterson et al., 2006). Further details of imputation and quality control have been published elsewhere (Eszlari et al., 2019).

### 2.4 Statistical analysis

We used the stable 1.26.0 Genome‐wide Complex Trait Analysis (GCTA) version for the estimation of SNP heritability. A minor allele frequency (MAF) cut‐off of 0.05 was used for all SNPs passing quality control measures, yielding 2,550,710 SNPs in the Budapest and 2,744,431 SNPs in the Manchester sample. We did not apply further LD pruning because GCTA by design accounts for linkage disequilibrium (Yang et al., 2016).

Polygenic scores were constructed using PRSice‐2 (2.3.3) and publicly available summary SNP effect size data (Lee et al., 2018), downloaded from https://www.thessgac.org/data. We used the effect sizes which were constructed without 23andMe data but released for all SNPs. A MAF threshold of 0.01, a clumping threshold of *R* ^2^ = 0.1 and a clumping window of 500 kb was used. For clumping, we used summary statistics from the Lee et al. GWAS and the pooled Budapest and Manchester samples to calculate LD. MAF thresholds were based on published recommendations (Coleman et al., 2016). We used the PGS constructed with the GWAS *p*‐value threshold that had the strongest association with a specific phenotype, highest educational level within the pooled sample. The best performance was achieved by the PGS with a *p*‐value threshold of 0.032, consisting of 18,261 SNPs. We used this PGS in all subsequent analyses. The performance of PGSs calculated using different *p*‐value thresholds is illustrated in Figure S1. All other calculated alternative PGSs were also significantly associated with educational level (*p*_max_ = 2.2 × 10^–6^). In total, we calculated 1340 PGSs with various *p*‐value inclusion thresholds, the mean correlation between all possible pairs of these was *r* = 0.902 (SD = 0.08).

We estimated PGS effect sizes as the point biserial (college completion) or Pearson point‐moment (years in education) correlation between the educational attainment phenotype and the PGS. We ran additional multivariate models controlling for the effects of age, sex, the first 10 genomic principal components and self‐reported psychiatric or pain‐related diagnoses. These were operationalized as generalized linear models with the fitglm() function in MATLAB 2018a specifying a binomial distribution and logit link (logistic regression for college completion) or a normal distribution and an identity link (linear regression for years in education). For logistic regressions, the PGS effect size was expressed as the Nagelkerke *R* ^2^ statistic, while for linear regressions it was expressed as multiple regression coefficients.

Because the variance of years in education was not equal in all subsamples, we corrected correlation coefficients for restriction of range using the formula by Schmidt and Hunter (2014) using the total Manchester sample as the reference.

We ran both GCTA and PGS analyses both with and without controlling for the first 10 genomic principal components.

## 3 RESULTS

### 3.1 PGS effects

The EA3 PGS was significantly associated with both categorical and continuous educational attainment phenotypes in both the total Budapest (*r*_college completion_ = 0.11 [*R* ^2^ = 0.012], *r*_years_ = 0.12 *R* ^2^ = 0.072) and Manchester (*r*_college completion_ = 0.22 [*R* ^2^ = 0.047], *r*_years_ = 0.22 [*R* ^2^ = 0.046]) samples (all *p* < .001). In multivariate models, we explored the effect age, sex, the first 10 genomic PCs and self‐reported illness (depression, suicidal attempt, manic disorder, anxiety disorder, obsessive‐compulsive disorder, schizophrenia, eating disorder, drug, or alcohol‐related disorder and/or pain‐related problems) on the PGS‐phenotype association (Tables S1 and S2). The inclusion of these covariates (especially age, sex, and genomic PCs) caused little change in effect sizes. Using ISCED‐derived years in education instead of the original records also did not substantially affect effect sizes (across all models Δ*β*_mean_ = 0.013, Δ*β*_SD_ = 0.018, absolute Δ*β*_Max_ = 0.054). The largest change was observed in the Manchester PostC age group, where the effect size increased by about 0.05; however, even this remained within a single standard error from the original value. In other subgroups, the change was negligible (Δ*β*_mean_ = 0.008).

**FIGURE 1:**
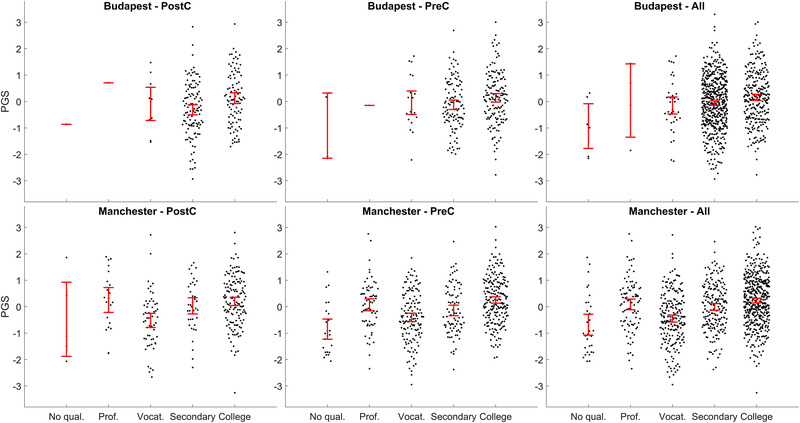
PGSs (shown as z‐scores) by country and educational levels. PostC and PreC indicate age groups, see Table 1 for details and definitions of educational levels. “All participants” includes participants with no age data. Whiskers indicate 95% confidence intervals (CIs) of the mean, overplotted with raw data. Note that some Budapest groups were represented by a single participant which did not permit the estimation of CIs and instead only the value is shown

Figure 1 shows group means and the dispersion of PGSs by country, age group, and educational attainment level. The highest mean values were always found in participants with tertiary education. Participants with secondary education had lower means, followed by individuals with vocational educations (O‐levels in the Manchester sample). Individuals with professional educations had somewhat higher means. We note, however, that the low number of participants with low educational attainments led to less precision in estimating mean PGSs. Note especially the limited educational attainment variability in the Budapest sample, with most participants having either high school or university educations.

**FIGURE 2:**
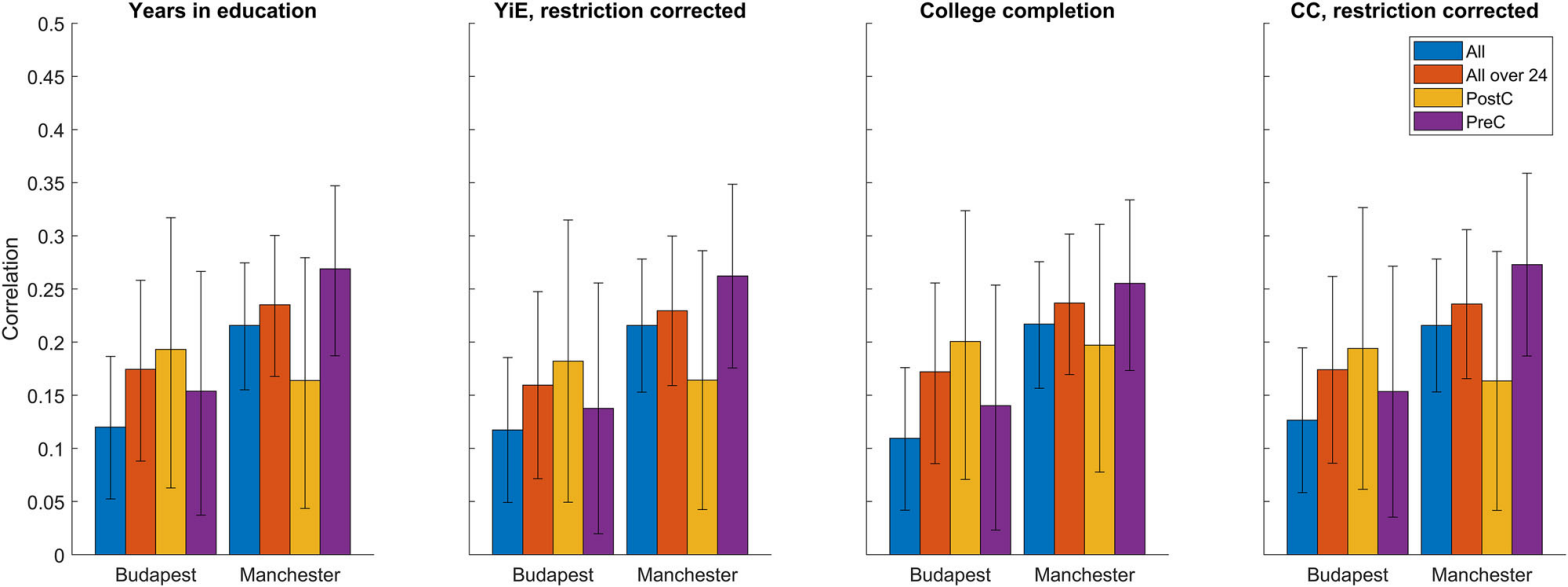
Associations between the best‐fit EA3 polygenic score and educational attainment by sample and age group. For college completion, the effect size is a point‐biserial correlation and for years in education, the effect size is a Pearson correlation coefficient. Error bars show 95% CIs. “Restriction corrected” refers to a PGS‐phenotype correlation corrected for restriction of range. PostC: participants at most 16 years old at FoC and at least 24 years old during data collection. PreC: participants at least 16 years old at FoC. “All over 24 years” refers to pooled PostC and PreC subsamples. “All” also includes participants younger than 24 years old at data collection and those with no age data.

Because the inclusion of covariates had little effect on the effect sizes, we illustrate the simple zero‐order point biserial (college completion) or Pearson (years in education) correlations in Figure 2. We show both correlations both with and without correcting for restriction of range, separately by sample and age group. The PGS was significantly associated with both phenotypes in all individual subsamples (*r* = 0.11‐0.27, *p* = <.001–.097).

We next estimated whether the PGS‐phenotype associations were different by age group. We excluded the youngest participants (age <24) from these analyses because of the low variability of educational attainment in this subgroup (see Table 1). With the continuous phenotype (years in education) as the dependent variable, the PGS*age group interaction was not statistically significant in either the Budapest (*F*_1,496_ = 0.05, *p* = .81) or the Manchester (*F*_1,770_ = 2.98, *p* = .08) samples. In order to assess age group and country effects on the relative strength of PGS effects, we compared effect sizes of each phenotype (college completion, years in education, both also corrected for restriction of range) between all subgroups using Fisher's *r*‐to‐*z* method (Rimfeld et al., 2018). No subgroup difference was significant after correcting for multiple comparisons. A trend (several nominally significant differences) for higher effect sizes in Manchester subsamples compared to the total Budapest sample was seen. In line with previous Estonian results, PostC Budapest subsamples had higher effect sizes than the PreC subsamples for all phenotypes, but this did not reach statistical significance at our sample size (*p*_min_ = 0.45). We report detailed results in Table S3.

We set the age cut‐off between PreC and PostC groups at 32 years in 2004–2005 (16–17 years old at FoC) following Rimfeld et al., but still somewhat arbitrarily. In order to test the effect of different age cut‐offs on the results, we performed a specification curve analysis using all possible PreC/PostC age cut‐offs between 26–45 years in 2004–2005 (Figure S2). In line with previous analyses, we never included those under 24 years old in either the PreC or the PostC groups. For both educational level and years in education, the trend of higher PGS effect sizes in the Budapest PostC sample persisted for all cut‐offs less than 40 years (birth year: 1964/65, at least 25 years old at FoC) with no similar effect in the Manchester sample. However, at this sample size, no age cut‐off yielded a statistically significant difference between the effect sizes in the PreC and PostC subsamples for either phenotype and sample.

### 3.2 SNP heritability

Genomic‐relationship‐matrix restricted maximum likelihood (GREML‐GCTA) SNP heritabilities indicated that in the Budapest sample, all common SNPs accounted for 34.4% (SE = 24%, *p* = .06) of the variance of years in education. In the Manchester sample, the same SNP heritability was 20.5% (SE = 20%, *p* = .13). Controlling for the first 10 genomic PCs, the values were *h* ^2^_SNP_ = 42.6% (Budapest, SE = 24.6%, *p* = .03) and *h* ^2^_SNP_ = 20.2% (Manchester, *p* = .15). In case of the college completion binary outcome, all common SNPs accounted for 52% (SE = 24%, *p* = .01) of the variance in the Budapest sample (53%, SE = 24.3%, *p* = .01 controlling for genomic PCs) and 40% (SE = 20.3%, *p* = .02) in the Manchester sample (36%, SE = 20.8%, *p* = .03 controlling for genomic PCs). Note that these estimates had wide confidence intervals due to the limited sample size. However, as our sample sizes were below the several thousand individuals usually recommended for this type of analysis (Knopik et al., 2016), and because GCTA models failed to properly converge when we further restricted samples to single age groups due to very low sample sizes, we did not perform SNP heritability analyses within these separately.

## 4 DISCUSSION

Ours is the first study to estimate molecular genetic effects on educational attainment in Hungary, and the second to do so in a former Warsaw Pact country. We are also unaware of any other behavior genetic study about educational attainment or cognitive functions in Hungarians, except from some data from the Hungarian Twin Registry published in a recent meta‐analysis (Silventoinen et al., 2020). The main goal of our study was to demonstrate the predictive performance of the EA3 PGS in a novel country. Despite its limited size, our sample was well‐powered for this purpose.

In our main analysis, we found that—in line with international results—the genetic variants discovered by a recent GWAS to predict educational attainment in Western European and American validation samples also do so in Hungary.

We compared findings in Hungarians to analogous results from an English comparison sample with identical recruitment protocols and phenotypic measurements. Once restriction of range was corrected for either statistically or by excluding very young participants presumably still in education, PGS effect sizes were not significantly lower in the Budapest sample.

An exact comparison of our PGS effect sizes with other studies is not feasible due to between‐study differences in genotyping, polygenic score construction (such as differences in the source GWAS and the selection of *p*‐value and MAF thresholds) and phenotype quality (including the specific phenotype used and its variance). However, we note that that the effect sizes in the Manchester sample were in line with those reported by independent studies using PGSs based on the same GWAS with more representative British and American datasets with higher quality phenotypes (Allegrini et al., 2019; Lee et al., 2018) including educational attainment and cognitive performance. The effect sizes in the Budapest sample were generally not substantially weaker than this. In sum, the relative strength of genetic effects in our Budapest sample were in line with those reported from other countries.

Our first auxiliary analysis aimed to replicate previous Estonian findings about the larger relative role of genetic effects after FoC. Because this previous finding established a clear prior hypothesis about the presence and the direction of this effect, we attempted these auxiliary analyses despite limitations in statistical power. Our replication was only partially successful. While we found substantially higher effect sizes for educational attainment phenotypes in the FoC subsamples in the Budapest, but not the Manchester sample, these differences did not reach statistical significance. Changes in educational policy surrounding FoC were similar in Hungary to Estonia (Hrubos et al., 2016; Ladányi, 1995). The previous Estonian study on the same effects (Rimfeld et al., 2018) invokes increases in educational meritocracy—first of all, the abandonment of political considerations in university admissions—as the chief driver of increased PGS effect sizes after FoC. Our results do not exclude the possibility of a similar change taking place in Hungary, but better powered genetic databases will be required for a conclusive replication.

Age, cohort, or country differences in the heritability of social traits can reflect mechanisms other than genuine historical societal differences. Differences in sampling bias is an especially strong candidate mechanism of creating spurious heritability differences. We were able to account for two sources of sampling bias: educational attainment variance and psychiatric illness. If study participants are recruited from a narrower educational attainment range in certain subgroups, heritability in that age group is biased downward. We eliminated this bias by controlling for restriction of range. If psychiatric disorders affect heritability and individuals in psychiatric disorders are oversampled in certain subgroups, estimated heritability in that subgroup will also be affected. We demonstrated that self‐reported psychiatric illness does not affect SNP heritability in the NewMood sample, therefore, subgroup differences in psychiatric illness are unlikely explanations of SNP heritability differences. We emphasize that we were unable to account for all possible sources of sampling bias (and other biases), warranting further caution about the results. We note that although due to limitations in statistical power, we limited our auxiliary analyses to replication instead of discovery and thus we were mainly interested in age group differences in the Budapest sample, the PreC‐PostC difference in the Manchester sample was even larger (with an opposite sign), even though no major historical change took place in England at the time of FoC in Central Europe.

Our second auxiliary analysis estimating SNP heritability in the Budapest subsample suggests that in line with recently published pedigree‐based results (Silventoinen et al., 2020) a substantial proportion of educational attainment variance is accounted for by common genetic variants in Hungarians, but once again our estimates are imprecise due to limited power and require replication in a larger sample.

Our work suffers from a number of limitations. The largest of these is the modest size of our sample, which allowed us to conclusively demonstrate the association of the PGS polygenic score with actual educational attainment in Hungarians, but limited statistical power to detect age and country effects on SNP heritability (Table S3). Systematically higher PGS effect sizes in the FoC Budapest sample suggest that a historical gene‐environment interaction may have taken place in Hungary during FoC, but this requires replication in larger samples. We note, however, that large, approximately population‐representative genetic databases like the Estonian Biobank are rare in Central and Eastern Europe, and therefore ours is probably the best Hungarian dataset currently available to test our hypotheses.

Second, our database was not nationally representative and most educational attainment differences existed between completion and non‐completion of college. This may have exerted a downward bias on our PGS predictive performance estimates due to range restriction, especially in the Hungarian sample. We corrected our results for restriction of range, but only relative to the subsample with the highest variance.

Third, a general limitation to between‐family molecular genetic studies is that they may reveal shared environmental instead of true, biological genetic effects through gene‐environment correlation (Young, 2019). On the one hand, SNPs used to construct PGSs or the relatedness matrix for GCTA may be associated with educational attainment because they index membership in families which influence educational attainment through cultural rather than genetic effects (residual stratification or “dynastic” effects (Morris et al., 2019)). On the other hand, SNPs may have a causal effect on parental phenotypes, which in turn influence offspring educational attainment (genetic nurture (Kong et al., 2018)). While both effects are known to operate and inflate PGS effect sizes in studies of unrelated individuals (Kong et al., 2018; Young, 2019; Young et al., 2018), predictive performance in within‐family studies (Belsky et al., 2018; Domingue et al., 2015; Selzam et al., 2019) demonstrates that a substantial portion of PGS effects reflects actual genetic influences. While we do not expect the effect of genetic nurture to be substantially different in Hungary than in other countries, we expect residual stratification to inflate PGS effect sizes more in our Manchester sample and other British between‐family studies than in our Budapest sample. This is because the GWAS, based on which we constructed our PGSs, contained substantial British study populations, but no Hungarians and a very limited number of participants from neighboring countries (specifically, 777 Austrians and 842 Croatians) which would bias stratification effects towards patterns which exist in Britain. Future Hungarian within‐family studies may provide a formal test of this hypothesis.

Finally, the investigated after‐FoC period ends in 2004–2005 at the time of data collection and our study does not investigate educational attainment after this time.

## 5 CONCLUSION

In sum, our work demonstrates that genetic variants discovered in international GWAS samples also predict educational attainment in Hungary with equal or only slightly reduced strength relative to an English sample. In line with Estonian data, individual genetic differences played a somewhat larger role shaping educational attainment in those graduating after the fall of Communism, but due to limitations in statistical power a more conclusive replication of this effect is needed. Similar findings from Hungary had not been previously available, and the results are likely of interest to those studying the society of Hungary and may serve as a model for other countries of the region without their own genetic studies.

## CONFLICT OF INTEREST

Bill Deakin has share options in P1vital. He has also performed speaking engagements, research, and consultancy for AstraZeneca, Autifony, Bristol‐Myers Squibb, Eli Lilly, Janssen‐Cilag, P1vital, Schering Plough, and Servier (all fees paid to the University of Manchester to reimburse them for the time taken). All the other authors declare no conflict of interest.

### PEER REVIEW

The peer review history for this article is available at https://publons.com/publon/10.1002/brb3.2430.

## Supporting information



SUPPORTING INFORMATION

## Data Availability

An anonymized dataset containing PGSs, city, age, sex, and educational attainment data is available at: https://osf.io/dfg38/.
