# Supplementary material for: Genetic effects on educational attainment in Hungary
Source: Brain Behav. 2021 Nov 29;12(1):e2430. doi: 10.1002/brb3.2430 (PMC8785634; doi:10.1002/brb3.2430)
Supplement: Supplementary file 1 — SUPPORTING INFORMATION [file BRB3-12-e2430-s001.docx]

**S1 Table. PGS effect sizes from logistic regression models with college graduation as a binary outcome.**

|  | **Budapest** | | | | **Manchester** | | | |
| --- | --- | --- | --- | --- | --- | --- | --- | --- |
|  | All | All over 24 | PostC | PreC | All | All over 24 | PostC | PreC |
| **No covariates** | 0.016 | 0.039 | 0.053 | 0.026 | 0.065 | 0.078 | 0.052 | 0.093 |
| **Age, sex** | 0.025 | 0.032 | 0.069 | 0.027 | 0.050 | 0.066 | 0.032 | 0.097 |
| **+PCs** | 0.014 | 0.039 | 0.058 | 0.025 | 0.056 | 0.073 | 0.048 | 0.091 |
| **+Disorders** | 0.013 | 0.036 | 0.052 | 0.017 | 0.049 | 0.067 | 0.041 | 0.083 |

This table summarizes PGS effect sizes (Nagelkerke R^2^) from logistic regression models using PGS as the predictor. In the second through fourth rows, the PGS effect size is the incremental Nagelkerke R^2^ (improvement in model fit) in models using also using the PGS as a predictor in addition to age and sex; age, sex and the first 10 genomic PCs; and age, sex, the first genomic PCs and self-reported pathology, respectively. Effect sizes are reported for sample (Budapest, Manchester) and age (all participants, all participants at least 24 years of age, PostC, PreC) specifications separately.

**S2. Table.** **PGS effect sizes from linear regression models with years in education as a continuous outcome.**

|  | **Budapest** | | | | **Manchester** | | | |
| --- | --- | --- | --- | --- | --- | --- | --- | --- |
|  | All | All over 24 | PostC | PreC | All | All over 24 | PostC | PreC |
| **No covariates** | 0.122 (0.04) | 0.190 (0.05) | 0.198 (0.07) | 0.176 (0.07) | 0.214 (0.03) | 0.243 (0.04) | 0.157 (0.06) | 0.285 (0.05) |
| **Age, sex** | 0.105 (0.03) | 0.190 (0.05) | 0.208 (0.07) | 0.185 (0.07) | 0.203 (0.03) | 0.241 (0.04) | 0.156 (0.06) | 0.291 (0.04) |
| **+PCs** | 0.112 (0.04) | 0.196 (0.05) | 0.215 (0.07) | 0.177 (0.07) | 0.204 (0.03) | 0.239 (0.04) | 0.153 (0.06) | 0.291 (0.05) |
| **+Disorders** | 0.107 (0.04) | 0.185 (0.05) | 0.211 (0.07) | 0.141 (0.07) | 0.194 (0.03) | 0.234 (0.04) | 0.145 (0.06) | 0.284 (0.05) |

This table summarizes PGS effect sizes (regression coefficients expressed in within-sample standard deviation units) from linear regression models using PGS as the predictor. In the second through fourth rows, the PGS effect size is the multiple regression in models using also using the PGS as a predictor in addition to age and sex; age, sex and the first 10 genomic PCs; and age, sex, the first genomic PCs and self-reported pathology, respectively. Effect sizes are reported for sample (Budapest, Manchester) and age (all participants, all participants at least 24 years of age, PostC, PreC) specifications separately. Standard errors are reported in parentheses.

**S3 Table. Age and country differences in the PGS effect sizes.**

|  | **Years in education** | | | | | | | | |
| --- | --- | --- | --- | --- | --- | --- | --- | --- | --- |
|  |  | **BP all** | **BP all 24** | **BP PostC** | **BP PreC** | **MAN All** | **MAN All 24** | **MAN PostC** | **MAN PreC** |
|  | **BP all** |  | 0.327 | 0.324 | 0.619 | **0.037** | **0.017** | 0.529 | **0.006** |
|  | **BP all 24** | 0.980 |  | 0.811 | 0.779 | 0.437 | 0.270 | 0.888 | 0.114 |
|  | **BP PostC** | 0.986 | 0.239 |  | 0.655 | 0.755 | 0.567 | 0.742 | 0.322 |
|  | **BP PreC** | 0.497 | 0.280 | 0.447 |  | 0.349 | 0.229 | 0.906 | 0.106 |
|  | **MAN all** | **2.085** | 0.778 | 0.312 | 0.937 |  | 0.670 | 0.443 | 0.298 |
|  | **MAN all 24** | **2.381** | 1.102 | 0.572 | 1.204 | 0.426 |  | 0.301 | 0.525 |
|  | **MAN PostC** | 0.629 | 0.141 | 0.329 | 0.118 | 0.768 | 1.033 |  | 0.148 |
|  | **MAN PreC** | **2.763** | 1.581 | 0.990 | 1.615 | 1.040 | 0.636 | 1.448 |  |
| **Years in education, corrected for restriction of range** | | | | | | | | | |
|  |  | **BP all** | **BP all 24** | **BP PostC** | **BP PreC** | **MAN All** | **MAN All 24** | **MAN PostC** | **MAN PreC** |
|  | **BP all** |  | 0.448 | 0.383 | 0.766 | **0.032** | **0.020** | 0.501 | **0.007** |
|  | **BP all 24** | 0.758 |  | 0.774 | 0.766 | 0.291 | 0.205 | 0.949 | 0.087 |
|  | **BP PostC** | 0.871 | 0.287 |  | 0.615 | 0.642 | 0.518 | 0.841 | 0.297 |
|  | **BP PreC** | 0.298 | 0.297 | 0.504 |  | 0.238 | 0.175 | 0.753 | 0.082 |
|  | **MAN all** | **2.143** | 1.057 | 0.466 | 1.181 |  | 0.760 | 0.446 | 0.365 |
|  | **MAN all 24** | **2.319** | 1.268 | 0.646 | 1.358 | 0.305 |  | 0.344 | 0.541 |
|  | **MAN PostC** | 0.674 | 0.064 | 0.200 | 0.315 | 0.762 | 0.946 |  | 0.178 |
|  | **MAN PreC** | **2.682** | 1.710 | 1.042 | 1.741 | 0.906 | 0.611 | 1.347 |  |
| **College completion** | | | | | |  |  |  |  |
|  |  | **BP all** | **BP all 24** | **BP PostC** | **BP PreC** | **MAN All** | **MAN All 24** | **MAN PostC** | **MAN PreC** |
|  | **BP all** |  | 0.260 | 0.219 | 0.652 | **0.019** | **0.008** | 0.207 | **0.007** |
|  | **BP all 24** | 1.125 |  | 0.715 | 0.665 | 0.396 | 0.239 | 0.735 | 0.163 |
|  | **BP PostC** | 1.230 | 0.365 |  | 0.493 | 0.819 | 0.620 | 0.968 | 0.473 |
|  | **BP PreC** | 0.451 | 0.433 | 0.686 |  | 0.245 | 0.153 | 0.500 | 0.108 |
|  | **MAN all** | 2.345 | 0.850 | 0.229 | 1.162 |  | 0.664 | 0.766 | 0.453 |
|  | **MAN all 24** | 2.635 | 1.178 | 0.496 | 1.428 | 0.435 |  | 0.562 | 0.726 |
|  | **MAN PostC** | 1.261 | 0.339 | **0.040** | 0.675 | 0.297 | 0.580 |  | 0.419 |
|  | **MAN PreC** | 2.703 | 1.394 | 0.717 | 1.609 | 0.750 | 0.351 | 0.808 |  |
| **College completion, corrected for restriction of range** | | | | | | | | | |
|  |  | **BP all** | **BP all 24** | **BP PostC** | **BP PreC** | **MAN All** | **MAN All 24** | **MAN PostC** | **MAN PreC** |
|  | **BP all** |  | 0.392 | 0.361 | 0.693 | 0.051 | **0.023** | 0.595 | **0.006** |
|  | **BP all 24** | 0.857 |  | 0.798 | 0.778 | 0.431 | 0.261 | 0.889 | 0.097 |
|  | **BP PostC** | 0.913 | 0.256 |  | 0.643 | 0.763 | 0.567 | 0.732 | 0.301 |
|  | **BP PreC** | 0.395 | 0.282 | 0.463 |  | 0.344 | 0.221 | 0.904 | 0.093 |
|  | **MAN all** | 1.949 | 0.787 | 0.301 | 0.946 |  | 0.658 | 0.439 | 0.261 |
|  | **MAN all 24** | 2.269 | 1.125 | 0.572 | 1.223 | 0.443 |  | 0.294 | 0.483 |
|  | **MAN PostC** | 0.532 | 0.140 | 0.343 | 0.120 | 0.773 | 1.049 |  | 0.131 |
|  | **MAN PreC** | 2.731 | 1.661 | 1.035 | 1.682 | 1.123 | 0.701 | 1.511 |  |

Identical tables are reported for all three phenotypes (educational level, years in education, years in education corrected for restriction of range). The upper diagonals contain z-values and the lower diagonal contain p-values for the relevant comparisons (bold denotes nominal significance at p-value<0.05). No difference was significant after correcting for multiple comparisons using the FDR method ([Benjamini and Hochberg, 1995](#_ENREF_7))). BP: Budapest sample, MAN: Manchester sample. PostC and PreC indicate age groups, see Table 1. „All” indicates all participants, including those with no age data, while „all 24” denotes all participants at least 24 years of age.


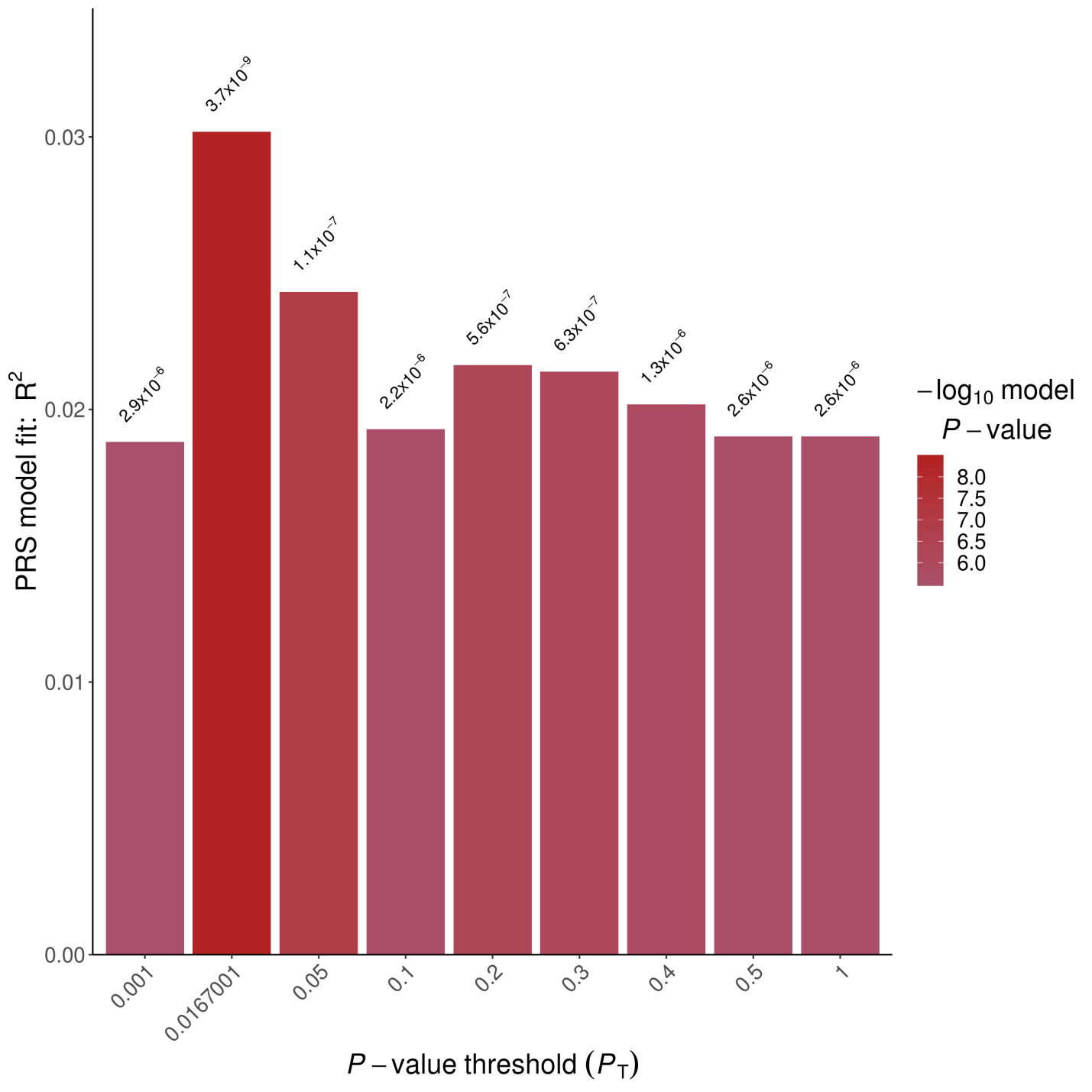


**S1 Fig. Educational level variance accounted for by PGSs constructed using the various p-value thresholds.** Thresholds are indicated on axis x. The best-performing PGS is indicated with its exact p-value thresholds. All PGS-phenotype associations are statistically significant (p_max_=2.2*10^-6^).


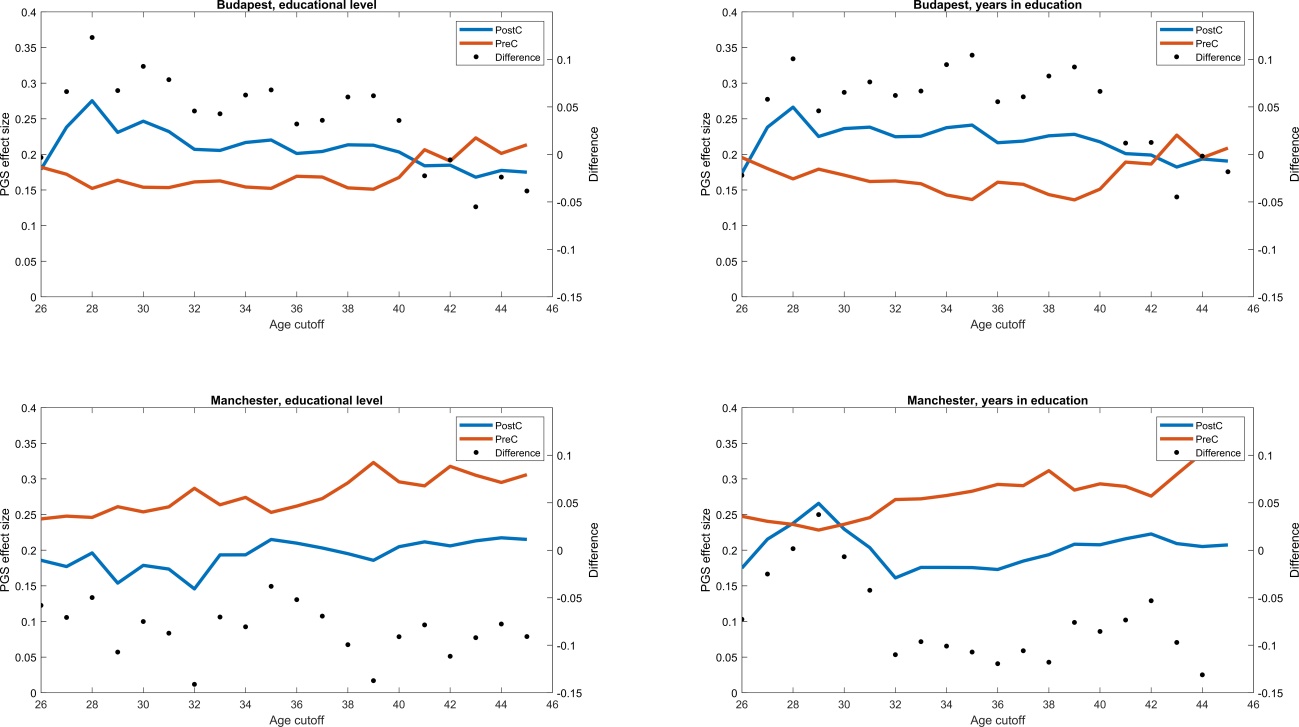


**S2 Fig. Specification curve analysis of the effect of age cutoff choice on PreC-PostC PGS effect size differences.** The lines indicate PostC and PreC effect sizes as a function of the age cutoff used to construct these categories (originally: 32 years) while dots indicate differences, shown on the right y axis. No PreC-PostC effect size pair differences reach statistical significance.
